# A genetic variant in long non-coding RNA MALAT1 associated with survival outcome among patients with advanced lung adenocarcinoma: a survival cohort analysis

**DOI:** 10.1186/s12885-017-3151-6

**Published:** 2017-03-03

**Authors:** Jian-Zhong Wang, Jing-Jun Xiang, Li-Ge Wu, Yan-Sen Bai, Zhuo-Wang Chen, Xiang-Qian Yin, Qing Wang, Wen-Hao Guo, Ying Peng, Huan Guo, Ping Xu

**Affiliations:** 1 Department of Oncology, Wuhan Iron and Steel (Group) Corporation Staff-Worker Hospital, 32 Qinghua Road, Wuhan, 430085 China; 2 Department of Occupational and Environmental Health and Ministry of Education Key Lab for Environment and Health, School of Public Health, Tongji Medical College, Huazhong University of Science and Technology, 13 Hangkong Rd, Wuhan, 430030 China

**Keywords:** Lung cancer, Survival, Long non-coding RNA, Metastasis-associated lung adenocarcinoma transcript 1 (MALAT1)

## Abstract

**Background:**

Recently studies have demonstrated that the long non-coding RNA (lncRNA) metastasis associated lung adenocarcinoma transcript 1 (MALAT1) may participate in the development and progression of lung cancer. In this study, we hypothesized that genetic variant of this lncRNA may affect the prognosis of lung cancer patients.

**Methods:**

We conducted a follow-up study for 538 patients with non–small cell lung carcinoma (NSCLC), including 140 early-staged (stage I and II) and 398 advanced staged (stage III and IV) patients. The genetic variant rs3200401 in MALAT1 was then genotyped among this population by using TaqMan assay. The association of this variant with overall survival of these patients was further analyzed.

**Results:**

It was shown that among the advanced lung adenoma patients, subjects carrying rs3200401 CT and CT + TT genotypes had significantly longer median survival time (MST = 29.9, 28.9 vs. 19.3 month, Long-rank *P* = 0.019 and 0.024, respectively) and decreased death risks [crude HR (95% CI) = 0.65 (0.43–0.98) and 0.64 (0.44–0.95), *P* = 0.040 and 0.025, respectively], when compared to subjects wtih the MALAT1 rs3200401 CC genotype. However, the beneficial effect of rs3200401 was not seen among early NSCLC and advanced lung squamous cell carcinoma patients. We further tested the TCGA data, and found that a higher expression of MALAT1 was associated with metastatic of advanced lung adenocarcinoma but not with lung squamous cell carcinoma.

**Conclusions:**

The rs3200401 T allele located on the lncRNA MALAT1 was associated with a better survival for advanced lung adenocarcinoma patients, which may offer a novel prognostic biomarker for this patient subgroup. However, these results need to be validated in larger populations of lung cancer and the biological function of this variant still warrants further investigation.

**Electronic supplementary material:**

The online version of this article (doi:10.1186/s12885-017-3151-6) contains supplementary material, which is available to authorized users.

## Background

Lung cancer was the most common cancer and one of the leading cause of cancer-related death worldwide, and it contributed to 13.0% of new cancer cases diagnosed in 2012 [1]. The non-small cell lung cancer (NSCLC) represents almost 85 to 90% of total diagnosed lung cancer, while lung adenocarcinoma is the most common histological subtype. The 5-year survival rate of advanced NSCLC is less than 5% based on the Surveillance, Epidemiology, and End Results (SEER) Program data [2].

Long non-coding RNAs are RNA genes larger than 200 bps, which do not code for proteins but regulate gene expression and protein synthesis [3]. Several lncRNAs were reported to be associated with tumorigenesis, tumor progression, and tumor metastasis [4–7]. In general, most lncRNAs are expressed at low levels. Metastasis associated lung adenocarcinoma transcript 1 (MALAT1), also known as noncoding nuclear-enriched abundant transcript 2 (NEAT2), is one of the most abundant and highly conserved lncRNAs, indicating its potential functional importance [8]. MALAT1 is broadly expressed in normal human tissues and overexpressed in numerous cancers as well as NSCLC [9]. It has been proposed that MALAT1 can regulate gene expression and alternative splicing. MALAT1 is localized in nucleus speckles at SRSF2 splicing domain in several cell lines and can interact with pre-mRNA-splicing factor SF2/ASF and CC3 antigen [10–14]. In vitro studies revealed that MALAT1 can regulate cell proliferation, migration, and vessel growth [15–17].

MALAT1 was originally identified as a marker for predicting metastasis and prognosis of early-staged NSCLC patients [9, 18]. Many researches also indicated that MALAT1 is linked to other cancer types or diseases as a negative prognosis factor, such as glioma, pancreatic cancer, colorectal cancer, etc. [19–25]. However, the associations between genetic polymorphisms of lncRNA MALAT1 and lung cancer prognosis were less investigated.

In this study, we genotyped the single nucleotide polymorphism (SNP) rs3200401 located in lncRNA MALAT1 and aimed to investigate its association with the survival outcome of 398 advanced NSCLC patients.

## Methods

### Study patients

The study enrolled 538 patients who were diagnosed as NSCLC and treated at the Department of Oncology at Wuhan Iron and Steel (Group) Corporation Staff-Worker Hospital between January 2003 and December 2012. Patients who were still alive on December 31, 2013 (132 patients) were considered as censored, and the survival time for each patient was calculated from the date when they were confirmed diagnosed of lung cancer until the date of death or the last follow-up. The demographic data, lifestyle risk factors (e.g. smoking status, drinking), medical history, and clinical features were gathered by interview or from the patients’ medical records. A large part of study patients has been studied and reported in our previous study [26].

### SNP selection and genotyping

The NCBI dbSNP database were used to select the SNP of MALAT1 (https://www.ncbi.nlm.nih.gov/snp/). It was indicated by the dbSNP database that there are 16 SNPs located on MALAT1 gene with the MAF > 0.01, however, only rs3200401 had the MAF > 0.10 in all the 1000 Genome, the NHLBI “Grand Opportunity” Exome Sequencing Project (GO-ESP), and Exome Aggregation Consortium (ExAC) projects (https://www.ncbi.nlm.nih.gov/variation/view/) (Additional file 1: Table S1). Thus, to acquire adequate statistical power, the SNP rs3200401 was investigated in the present study, while the other SNPs with MAF < 0.10 were not selected. Genomic DNA samples were extracted from blood cells by using Gentra Puregene Blood Kit (QIAGEN, Hilden, Germany) following manufacturer’s instructions. The MALAT1 polymorphism, rs3200401 C > T, was genotyped by TaqMan assay among all study subjects using ABI 7900HT Sequence Detection System (Applied Biosystems, Waltham, Massachusetts, USA) and each sample was analyzed in duplicate. The primers and probes were purchased from Life Technologies (Catalog No. C___3246069_10). The genotyping call rate was 98.1% and the concordance rate was 100%.

### Statistical analysis

Kaplan-Meier analysis and log-rank test were used to assess the associations between survival time and demographic characteristics, clinical features, and MALAT1 rs3200401 genotypes. We use dominant model to assess the association of SNP rs3200401 genotypes and survival outcome of early and advanced NSCLC patients, respectively. The multivariate Cox regression models, with adjustment for age, sex, smoking status, histology, TNM stage, and therapy treatments of surgical resection, chemotherapy, and radiotherapy, were used to estimate the adjusted hazard ratio (HR) and 95% CIs for the effect of MALAT1 rs3200401 genotypes on death risk for NSCLC patients. All data analyses were performed in SPSS software (version 22, IBM SPSS Statistics, IBM Corporation, Chicago, IL). Power analysis was performed by using Power and Sample Size version 13.2 application in SAS (version 9.4, SAS Institute Inc., Cary, NC).

## Results

### Patient characteristics

The demographic information and clinical features for NSCLC patients were presented in Table 1. Among these patients, a total of 78 early-staged and 328 advanced NSCLC patients were confirmed death of lung cancer until the date of the last follow-up. Among these NSCLC patients, there are 450 males (84%) and 88 (16%) females, with a median age of 66.5 years (range, 40–87). Log-rank test and univariate cox-regression showed that NSCLC patients with age > 65, advanced stage (stage III or IV), and without surgical treatment had lower median survival time (MST) and higher death risks than their counterparts (all log-rank *P* < 0.05). However, there were no significant effects of smoking, chemotherapy, and radiotherapy on the MST and death risk of these NSCLC patients. The statistical power analysis showed that, as for the SNP rs3200401 (MAF = 0.187) analyzed in this study, it had the statistical power of 0.913 to detect the association with HR = 1.4 by using 398 subjects in the survival analysis.

**Table 1:** Demographic and clinical characteristics of 538 NSCLC patients

Variables	NSCLC Patients *n* (%)	Deaths *n* = 328	MST (months)	Log-rank *P*	Crude HR (95% CI)
Age					
≤65	253 (0.47)	178	29.9	---	1.00 (Reference)
>65	285 (0.53)	228	16.4	<0.001	1.54 (1.27–1.88)
Sex					
Male	450 (0.84)	345	20.7	---	1.00 (Reference)
Female	88 (0.16)	61	28.9	0.042	0.75 (0.57–0.99)
Smoking					
Never	93 (0.17)	65	30.5	---	1.00 (Reference)
Ever	445 (0.83)	341	21.1	0.11	1.24 (0.95–1.61)
Histology					
Adenocarcinoma	237 (0.44)	165	24.5	---	1.00 (Reference)
Squamous cell carcinoma	188 (0.35)	143	28.1	0.81	1.03 (0.82–1.29)
Others	113 (0.21)	98	13.3	<0.01	1.74 (1.35–2.23)
Clinical Stage					
Early	140 (0.26)	78	57.6	---	1.00 (Reference)
Advanced	398 (0.74)	328	17.9	<0.001	2.43 (1.89–3.12)
Surgery					
No	334 (0.62)	281	17.6	---	1.00 (Reference)
Yes	204 (0.38)	125	36.1	<0.01	0.53 (0.43–0.65)
Chemotherapy					
No	126 (0.23)	95	20.1	---	1.00 (Reference)
Yes	412 (0.77)	311	23.7	0.24	0.87 (0.69–1.1)
Radiotherapy					
No	291 (0.54)	208	22.9	---	1.00 (Reference)
Yes	247 (0.46)	198	21.5	0.24	1.08 (0.89–1.31)

Patients with age > 65 or with advanced staging had a significantly shorter survival time and higher risk of death when comparing with patients with age ≤ 65 or diagnosed at early stage [MST = 16.4 vs. 29.9 months, log-rank *P* < 0.001, HR (95% CI) = 1.54 (1.27–1.88); MST = 17.9 vs. 57.6 months, log-rank *P* < 0.001, HR (95% CI) = 2.43 (1.89–3.12), respectively] (Table 1). In addition, NSCLC patients who underwent surgical treatment had a longer survival time and a lower risk of death [MST = 36.1 vs.17.6 months, log-rank *P* <0.01, HR (95% CI) = 0.53 (0.43–0.65)] (Table 1). However, there were no significant associations of smoking status, chemotherapy, and radiotherapy (all log-rank *P* > 0.05) with the survival outcomes among NSCLC patients.

### Association of genetic variant rs3200401 with survival outcome among NSCLC patients

The associations between lncRNA MALAT1 rs3200401 genotypes and survival outcome of early-staged NSCLC patients were shown in Table 2. No significant associations were found between rs3200401 genotypes and the MST and death risk of early NSCLC patients, either in adenocarcinoma or squamous cell carcinoma. (Table 2).

**Table 2:** Associations between LncRNA MALAT1 rs3200401 genotypes and survival of early-staged NSCLC patients

Genotypes	Patients (%)	Deaths	MST (months)	Log-rank *P*	Crude HR (95% CI)	Crude *P*	Adjusted HR (95% CI)^a^	Adjusted *P*
All patients								
CC	86	48	55.3	--	1.00 (Reference)	---	1.00 (Reference)	---
CT	49	26	61.4	0.940	0.92 (0.57–1.48)	0.717	0.92 (0.56–1.51)	0.734
TT	3	2	39.2	0.980	1.15 (0.28–4.77)	0.848	1.07 (0.25–4.65)	0.929
CT + TT	52	28	61.4	0.760	0.93 (0.58–1.48)	0.756	0.93 (0.57–1.51)	0.760
Adenocarcinoma								
CC	37	18	62.9	--	1.00 (Reference)	---	1.00 (Reference)	---
CT	18	6	---	0.650	0.65 (0.25–1.64)	0.357	0.42 (0.15–1.19)	0.104
TT	1	0	---	---	---	---	---	---
CT + TT	19	6	---	0.290	0.61 (0.24–1.55)	0.297	0.40 (0.14–1.12)	0.082
Squamous cell carcinoma								
CC	41	25	48.6	--	1.00 (Reference)	---	1.00 (Reference)	---
CT	26	16	57.6	0.920	0.88 (0.46–1.65)	0.683	0.81 (0.40–1.64)	0.563
TT	1	1	41.2	0.880	1.63 (0.22–12.23)	0.635	1.58 (0.17–14.49)	0.685
CT + TT	27	17	57.6	0.740	0.90 (0.48–1.68)	0.743	0.83 (0.42–1.66)	0.602

^a^Adjusted for age, sex, smoking status, surgery, chemotherapy and radiotherapy

Among the advanced NSCLC patients, those carrying MALAT1 rs3200401 CT and rs3200401 CT + TT genotypes have significantly longer MST than those with rs3200401 CC genotype (MST = 22.6, 21.8 vs.15.9 months, and log-rank *P* = 0.045 and 0.034, respectively) (Table 3). The univariate cox proportional hazard models showed that, when compared to the rs3200401 CC genotype, the rs3200401 CT and CT + TT genotypes were associated with significant lower death risk for advanced NSCLC patients [crude HR (95% CI) = 0.78 (0.61–0.99) and 0.78 (0.61–0.98), *P* = 0.046 and 0.034, respectively]. But after adjusted by potential confounders, no statistically significant associations were found between CT [adjusted HR (95% CI) = 0.82 (0.64–1.05), *P* = 0.119] and CT + TT genotypes [adjusted HR (95% CI) = 0.81 (0.64–1.04), *P* = 0.094] with death risk of advanced NSCLC patients.

**Table 3:** Associations between LncRNA MALAT1 rs3200401 genotypes and survival of advanced NSCLC patients

Genotypes	Patients (%)	Deaths	MST (months)	Log-rank *P*	Crude HR (95% CI)	Crude *P*	Adjusted HR (95% CI)^a^	Adjusted *P*
All patients								
CC	259 (66.4)	219	15.9	---	1.00 (Reference)	---	1.00 (Reference)	---
CT	120 (30.8)	94	22.6	0.045	0.78 (0.61–0.99)	0.046	0.82 (0.64–1.05)	0.119
TT	11 (2.8)	8	21.8	0.353	0.71 (0.35–1.44)	0.345	0.73 (0.36–1.50)	0.392
CT + TT	131 (33.6)	102	21.8	0.034	0.78 (0.61–0.98)	0.034	0.81 (0.64–1.04)	0.094
Adenocarcinoma								
CC	119 (68.4)	99	19.3	---	1.00 (Reference)	---	1.00 (Reference)	---
CT	48 (27.6)	31	29.9	0.019	0.62 (0.41–0.93)	0.020	0.65 (0.43–0.98)	0.040
TT	7 (4.0)	5	19.1	0.804	0.87 (0.36–2.15)	0.767	0.89 (0.35–2.24)	0.806
CT + TT	55 (31.6)	36	28.9	0.024	0.65 (0.44–0.95)	0.025	0.64 (0.44–0.95)	0.025
Squamous cell carcinoma								
CC	76 (63.3)	62	17.9	---	1.00 (Reference)	---	1.00 (Reference)	---
CT	42 (35.0)	37	20.6	0.798	1.05 (0.70–1.59)	0.800	1.14 (0.75–1.75)	0.538
TT	2 (1.7)	2	11	0.999	1.01 (0.25–4.13)	0.992	1.46 (0.25–8.56)	0.676
CT + TT	44 (36.7)	39	20.6	0.805	1.05 (0.70–1.57)	0.805	1.05 (0.70–1.57)	0.805

^a^Adjusted for age, sex, smoking status, surgery, chemotherapy and radiotherapy

**Fig. 1:**
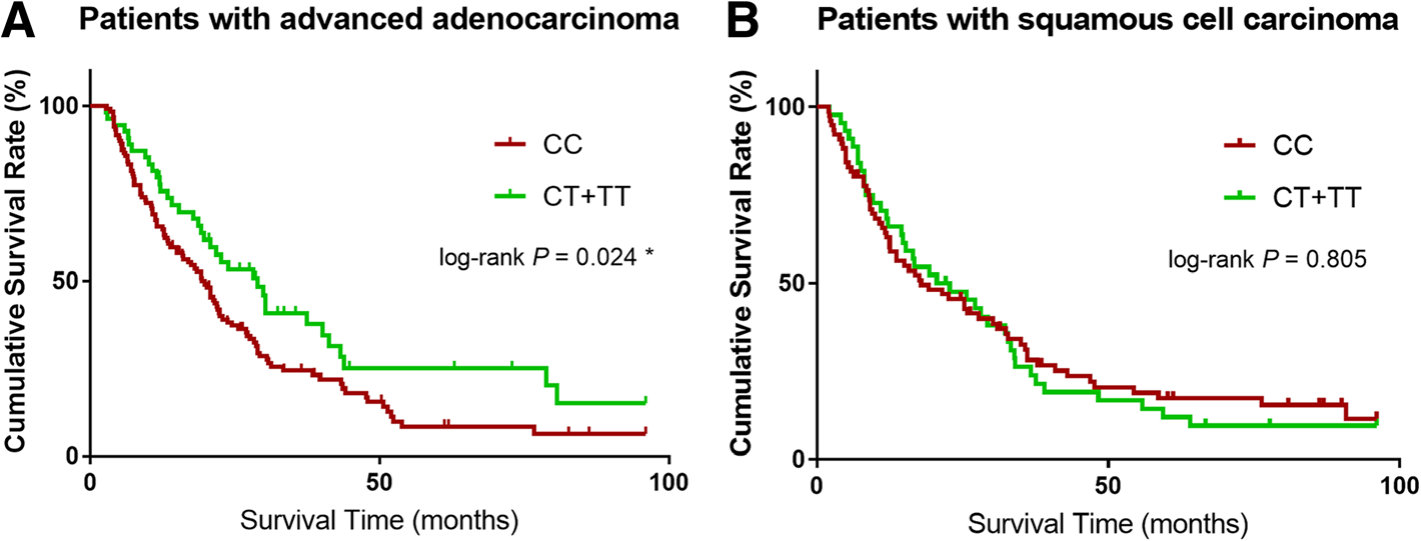
**a** Kaplan-Meier estimates for the overall survival of patients with advanced lung adenocarcinoma according to MALAT1 rs3200401 C > T. **b** Kaplan-Meier estimates for the overall survival of patients with advanced lung squamous cell carcinoma according to MALAT1 rs3200401 C > T

We further analyzed the associations of MALAT1 rs3200401 with survival outcomes among advanced patients with lung adenocarcinoma and squamous cell carcinoma separately (Table 3). Compared to advanced lung adenocarcinoma patients carrying the rs3200401 CC genotype, those with rs3200401 CT and CT + TT genotypes had significant longer MST and lower risk of death (MST = 29.9 and 28.9 months vs. 19.3 months, log-rank *P* = 0.019 and 0.024) [crude HR (95% CI) = 0.62 (0.41–0.93) and 0.65 (0.44–0.95); adjusted HR (95% CI) = 0.65 (0.43–0.98) and 0.64 (0.44–0.95), respectively] (Table 3) (Fig. 1a). However, this effect was not seen among advanced lung squamous cell carcinoma patients (Fig. 1b).

### Stratified analysis for advanced NSCLC patients

We then stratified the advanced lung adenocarcinoma and squamous cell carcinoma patients by age, sex, smoking, TNM stage, surgical operation, chemo- or radio-therapy, respectively (Table 4). We found the rs3200401 CT + TT genotype was associated with a decreased death risk at a borderline significance among lung adenocarcinoma patients with age ≤ 65 [HR (95% CI) = 0.59 (0.35–1.00), *P* = 0.054]) and stage IIIA-IIIB [HR (95% CI) = 0.54 (0.29–1.01), *P* = 0.053]. No significant associations between rs3200401 genotypes and survival were seen among squamous cell carcinoma patients in each stratum.

**Table 4:** Stratified analysis of lncRNA MALAT1 rs3200401 genotypes associated with advanced NSCLC patients’ survival

Variables	Adenocarcinoma		Squamous cell carcinoma	
	Adjusted HR (95% CI)^a^ CT + TT vs. CC	*P* ^a^	Adjusted HR (95% CI)^a^ CT + TT vs. CC	*P* ^a^
Age				
≤65	0.59 (0.35–1.00)	0.054	1.32 (0.69–2.53)	0.397
>65	0.94 (0.51–1.74)	0.850	0.95 (0.53–1.69)	0.862
Sex				
Male	0.66 (0.42–1.05)	0.081	1.04 (0.67–1.63)	0.857
Female	0.64 (0.26–1.54)	0.317	---	---
Smoking				
Never	0.44 (0.18–1.10)	0.079	16.73 (0.10–2963.81)	0.285
Ever	0.73 (0.46–1.15)	0.170	1.01 (0.65–1.60)	0.933
TNM Stage				
IIIA-IIIB	0.54 (0.29–1.01)	0.053	1.08 (0.65–1.79)	0.779
IV	0.83 (0.48–1.41)	0.484	0.91 (0.42–1.96)	0.810
Surgical operation				
No	0.69 (0.42–1.13)	0.137	0.99 (0.61–1.63)	0.982
Yes	0.56 (0.27–1.16)	0.118	1.12 (0.50–2.51)	0.792
Chemotherapy or Radiotherapy				
No	0.47 (0.05–4.55)	0.516	4.73 (0.35–63.42)	0.241
Yes	0.71 (0.47–1.07)	0.098	1.07 (0.70–1.65)	0.753

^a^Adjusted for age, smoking, TNM stage, surgery, chemo- and radio-therapy

## Discussion

In this follow-up study for case-only survival analysis, we investigated the association of the genetic variation rs3200401 in lncRNA MALAT1 with the survival outcome of NSCLC patients. We found that among advanced lung adenocarcinoma patients, those carrying MALAT1 rs3200401 CT or CT + TT genotypes had significant longer survival time and decreased death risks than those carrying rs3200401 CC genotype. This finding suggested that rs3200401 C > T variant of MALAT1 might be a potential prognostic biomarker for predicting the survival of advanced lung adenocarcinoma patients.

Metastasis is the major cause of death from lung cancer [27], and MALAT1 was significantly associated with metastasis of early-stage NSCLC patients [9]. The in vitro cell and in vivo animal studies had revealed that the expression level of lncRNA MALAT1 is related to cell migration potential and tumor growth. Increased MALAT1 expression in tumor tissues of NSCLC patients is associated with an unfavorable overall survival [19], while the high expression of MALAT1 in tumor tissues was also found to be associated with an increased risk of metastasis and a poor overall survival among colorectal cancer [21], pancreatic cancer [22], glioma [23], and clear cell renal cell carcinoma [24]. MALAT1 can induce metastasis through various mechanisms. The in vitro siRNA-mediated MALAT1 silencing resulted in impaired lung cancer cell motility by altering the expression levels of cell motility-related genes, such as HMMR at pre-mRNA transcriptional level and CTHRC1, CCT4 and ROD1 at post-transcriptional level [15]. Inhibition of MALAT1 was seen to have an anti-proliferative effect and controls phenotypic switch in endothelial cells, indicating that MALAT1 may regulate angiogenesis and result in metastasis [17, 28]. More researches suggested that upregulated MALAT1 can induce an epithelial-to-mesenchymal transition (EMT) and bladder cancer cell migration [29] and promote brain metastasis [30].

**Fig. 2:**
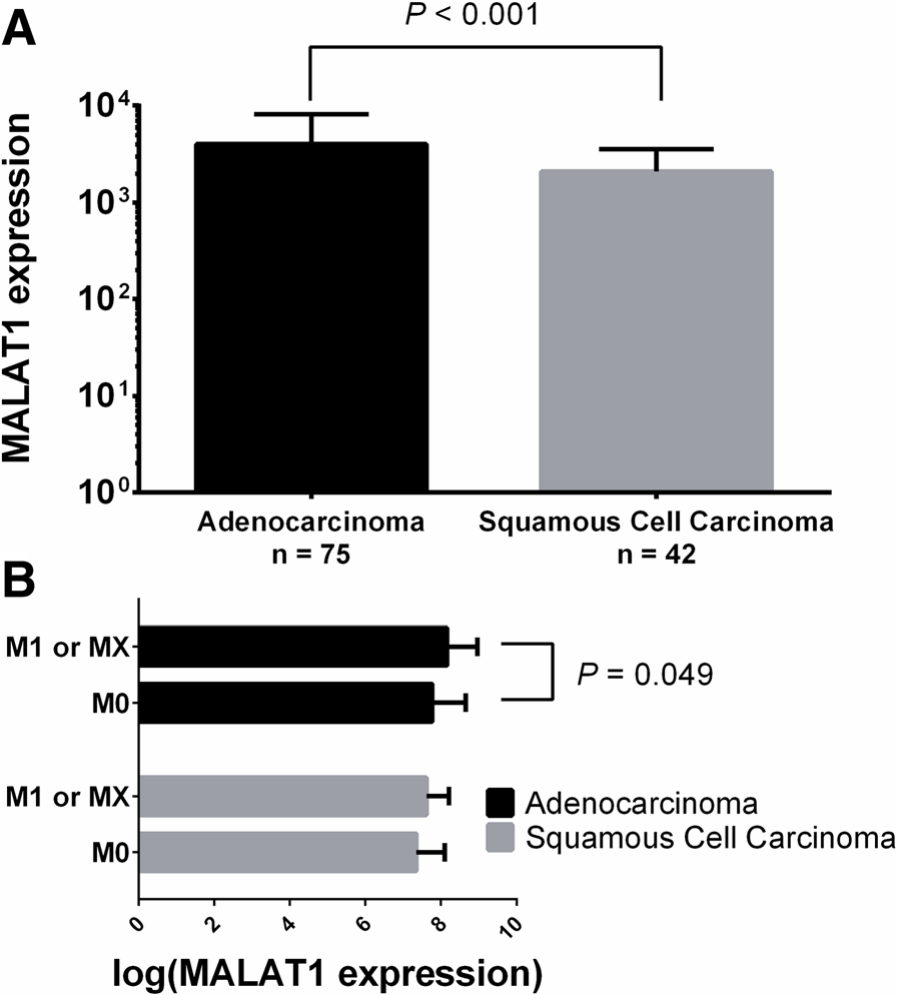
**a**. The different expression levels of MALAT1 between lung adenocarcinoma and squamous cell carcinama tissues; **b**. differences in tissues from M1 or Mx to M0 patients

Although MALAT1 expression in lung cancer tissue was reported to be associated with poor prognosis in lung squamous cell carcinoma [19], our findings indicated that the SNP rs3200401 cannot affect the survival outcome of lung squamous cell carcinoma patients. Ji et al. found that the association of MALAT1 with metastasis of NSCLC was distinct among different histological subtypes: MALAT1 expression in metastatic lung adenocarcinoma was several fold higher than in non-metastatic adenocarcinoma, but no significant differences were found between metastatic and non-metastatic lung squamous cell carcinoma patients [9]. We obtained the clinical data and MALAT1 gene expression data from the cancer genome atlas (TCGA) using cBioPortal [31]. A total of 654 patients with primary lung cancer (359 lung adenocarcinoma and 295 lung squamous cell carcinoma) and available MALAT1 expression data from white population were included. Among them, a total of 117 patients were diagnosed at an advanced stage. The demographic data for these advanced NSCLC patients was presented in Additional file 1: Table S2. We found significant higher MALAT1 expression levels in lung adenocarcinoma tissues than in lung squamous cell carcinoma tissues (*P* <0.001, Fig. 2a). In 75 tumors with advanced lung adenocarcinoma, higher expression levels of MALAT1 were seen in tissues from M1 or Mx patients than those from M0 patients (*P* = 0.049). However, this difference was not shown in 42 patients with advanced lung squamous cell carcinoma (Fig. 2b). Considering the different characteristics of these two major subtypes of NSCLC, the lncRNA MALAT1 may induce tumor metastasis through different mechanisms between adenocarcinoma and squamous cell carcinoma.

Regarding the biological function for lncRNA MALAT1, it was found to localize to nuclear speckles and interact with serine/arginine-rich (SR) proteins like serine/arginine-rich splicing factor 1 (SRSF1), SRSF2 (SC35) and RNA-binding protein with serine-rich domain 1 (RNPS1) that controls alternative splicing of pre-mRNA, a transcriptional level regulation of gene expression [10, 13, 32–35]. Large studies have reported that genetic polymorphisms on certain genes may affect the susceptibility of lung cancer [36, 37], sensitivity of chemo- or radiotherapy [38–40], and length of survival or prognosis [41–44]. Liu et al. found a borderline significant association between rs619586 in MALAT1 and decreased hepatocellular carcinoma risk [45]. Another study carried out by Gong et al. did not found any association between rs619586 genotype and lung cancer risk, but patients with rs619586 A allele had more chance of response to platinum-based chemotherapy [40]. In this study, we did not investigate the rs619586 of MALAT1 because the dbSNP database suggested it is a low-frequency SNP. Further studies with larger sample sized populations could investigate the low-frequency SNPs of MALAT1 with higher detection power, and the biological functions for the positive variants of MALAT1 also warrant further deep investigation.

The interaction between lncRNA and other molecules was probably determined by its structure rather than by its sequence. The polymorphism within lncRNA sequence may exert its function through alternative splicing of the transcript or lncRNA secondary structure change, resulting in gain or loss of function [46]. The SNP rs3200401 C > T variant locates in the region M of MALAT1 (6008–7011 nt), which is one of the binding sites to SRSF2 [34]. We use lncRNASNP database to predict potential functions of this SNP [47], such as structure change and miRNA-lncRNA interaction. We found that the C > T variation of rs3200401 caused 1.62 kcal/mol minimal free energy (MFE, ΔG) change, which may alter structural features of MALAT1 (Additional file 2: Figure S1), resulting in weaken interaction between MALAT1 and its binding protein SRSF2. MALAT1 can modulate phosphorylation of SRSF2, interact with SR proteins as a “molecular sponge” and influence their stability, and regulate the alternative splicing of pre-mRNAs [10, 13]. SRSF2 and phosphorylated SRSF2 were reported to correlate with aggressive features of lung adenocarcinoma but not with lung squamous cell carcinoma patients [48]. It was biologically possible that SNP rs3200401 C > T variant may cause MALAT1-SRSF2 binding loss, affect phosphorylation of SRSF2, down-regulate phosphorylation of SRSF2, change the alternative splicing of pre-mRNAs, and then alter the expression levels of metastasis associated genes. These effects may result in a lower aggressive feature and a better survival for lung adenocarcinoma but not for squamous cell carcinoma patients.

Although this is the first study to describe lncRNA SNP and lung adenocarcinoma survival, some limitations should be taken into consideration. First, this study used a single-institution cohort to investigate the association between MALAT1 variant and the survival outcome of lung cancer patients for practical reason. It would be ideal to have a multi-center based replication to validate our findings. Without such replication, our findings should be considered preliminary. Second, to get adequate statistical power by using this moderate cohort of lung cancer patients, we only choose the SNP with MAF > 0.1 (rs3200401) to investigate in the present study. The other low-frequency SNPs in MALAT1 were also needed to be investigated in further large sample-sized populations. Finally, it is a pity that there were no rs3200401 genotype data in the TCGA database, except for the expression of MALAT1 in lung tumor tissues. To give a clue for further interpretation and explanation of the biological function of rs3200401, we still analyzed the TCGA data and found that a higher MALAT1 expression level was associated with a worse survival outcome among advanced lung adenocarcinoma patients. But the biological function of this SNP and its effect on MALAT1 expression level need to be deep investigated by further biological studies.

## Conclusions

In conclusion, this study revealed that the genetic variation SNP rs3200401 T allele located on lncRNA MALAT1 was associated with a better survival of advanced lung adenocarcinoma patient, while this effect was not seen among lung squamous cell carcinoma patients. The protective effect of rs3200401 T allele may because it can influence the secondary structure of lncRNA MALAT1, or influence the interaction between MALAT1 and SR proteins thus by altering the expression levels of metastasis associated genes. The MALAT1 rs3200401 T allele may serve as a novel biomarker for predicting clinical outcomes of lung adenocarcinoma. Further large population based survival analysis and mechanistic studies are required to confirm our findings.

## Additional file

Additional file 1: Table S1. The SNPs located on the lncRNA MALAT1 gene (data source: the dbSNP database). **Table S2.** Demographic and clinical characteristics of 117 advanced NSCLC patients from TCGA. (DOCX 24.8 kb)
Additional file 2: Figure S1. Predicted secondary structures of lncRNA MALAT1. C > T variation of rs3200401 caused 1.62 kcal/mol minimal free energy (MFE, ΔG) change, which may alter structural features of MALAT1, resulting in weaken interaction between MALAT1 and its binding protein SRSF2. (TIF 215 kb)

## Abbreviations

lncRNA: Long non-coding RNA
MALAT1: Metastasis associated lung adenocarcinoma transcript 1
NSCLC: Non–small cell lung carcinoma
SNP: Single nucleotide polymorphism
