# Supplementary material for: A genetic variant in long non-coding RNA MALAT1 associated with survival outcome among patients with advanced lung adenocarcinoma: a survival cohort analysis
Source: BMC Cancer. 2017 Mar 3;17:167. doi: 10.1186/s12885-017-3151-6 (PMC5335789; doi:10.1186/s12885-017-3151-6)
Supplement: Additional file 1: Table S1. — The SNPs located on the lncRNA MALAT1 gene (data source: the dbSNP database). Table S2. Demographic and clinical characteristics of 117 advanced NSCLC patients from TCGA. (DOCX 24.8 kb) [file 12885_2017_3151_MOESM1_ESM.docx]

**Additional file 1: Table S1**. The SNPs located on the lncRNA MALAT1 gene (data source: the dbSNP database).

| **SNP ID** | **Location** | **1000G MAF** | **GO-ESP MAF** | **ExAC MAF** |
| --- | --- | --- | --- | --- |
| rs116042952 | [65,497,787](https://www.ncbi.nlm.nih.gov/variation/view/) | T=0.0190 | T=0.0145 | T=0.0041 |
| [rs11227209](https://www.ncbi.nlm.nih.gov/projects/SNP/snp_ref.cgi?rs=11227209) | [65,497,960](https://www.ncbi.nlm.nih.gov/variation/view/) | G=0.0112 | G=0.0002 | G=0.0041 |
| [rs619586](https://www.ncbi.nlm.nih.gov/projects/SNP/snp_ref.cgi?rs=619586) | [65,498,698](https://www.ncbi.nlm.nih.gov/variation/view/) | G=0.0661 | G=0.0290 | G=0.0580 |
| [rs150458267](https://www.ncbi.nlm.nih.gov/projects/SNP/snp_ref.cgi?rs=150458267) | [65,498,766](https://www.ncbi.nlm.nih.gov/variation/view/) | C=0.0192 | C=0.0154 | C=0.0039 |
| [rs77535011](https://www.ncbi.nlm.nih.gov/projects/SNP/snp_ref.cgi?rs=77535011) | [65,498,827](https://www.ncbi.nlm.nih.gov/variation/view/) | C=0.0144 | C=0.0131 | C=0.0034 |
| [rs11540779](https://www.ncbi.nlm.nih.gov/projects/SNP/snp_ref.cgi?rs=11540779) | [65,499,070](https://www.ncbi.nlm.nih.gov/variation/view/) | T=0.0126 | T=0.0009 | T=0.0061 |
| [rs11540782](https://www.ncbi.nlm.nih.gov/projects/SNP/snp_ref.cgi?rs=11540782) | [65,499,637](https://www.ncbi.nlm.nih.gov/variation/view/) | C=0.0194 | C=0.0098 | C=0.0049 |
| [rs60236485](https://www.ncbi.nlm.nih.gov/projects/SNP/snp_ref.cgi?rs=60236485) | [65,499,935](https://www.ncbi.nlm.nih.gov/variation/view/) | A=0.0437 |  | A=0.0047 |
| [rs7927113](https://www.ncbi.nlm.nih.gov/projects/SNP/snp_ref.cgi?rs=7927113) | [65,500,727](https://www.ncbi.nlm.nih.gov/variation/view/) | A=0.0439 | A=0.0384 | A=0.0107 |
| [rs79910129](https://www.ncbi.nlm.nih.gov/projects/SNP/snp_ref.cgi?rs=79910129) | [65,501,008](https://www.ncbi.nlm.nih.gov/variation/view/) | A=0.0232 | A=0.0266 |  |
| [rs664589](https://www.ncbi.nlm.nih.gov/projects/SNP/snp_ref.cgi?rs=664589) | [65,501,878](https://www.ncbi.nlm.nih.gov/variation/view/) | G=0.0695 | G=0.0332 | G=0.0615 |
| [rs1056816](https://www.ncbi.nlm.nih.gov/projects/SNP/snp_ref.cgi?rs=1056816) | [65,502,633](https://www.ncbi.nlm.nih.gov/variation/view/) | G=0.0176 | G=0.0342 | G=0.0292 |
| [rs73497102](https://www.ncbi.nlm.nih.gov/projects/SNP/snp_ref.cgi?rs=73497102) | [65,503,505](https://www.ncbi.nlm.nih.gov/variation/view/) | A=0.0435 | A=0.0384 | A=0.0106 |
| [rs115795653](https://www.ncbi.nlm.nih.gov/projects/SNP/snp_ref.cgi?rs=115795653) | [65,504,176](https://www.ncbi.nlm.nih.gov/variation/view/) | G=0.0136 | G=0.0128 | G=0.0037 |
| [rs3200401](https://www.ncbi.nlm.nih.gov/projects/SNP/snp_ref.cgi?rs=3200401) | 65,504,361 | T=0.1432 | T=0.1890 | T=0.1659 |
| [rs60151940](https://www.ncbi.nlm.nih.gov/projects/SNP/snp_ref.cgi?rs=60151940) | 65,504,912 | T=0.0537 | T=0.0493 | T=0.0139 |

**Abbreviation:** 1000G, 1000 Genome; GO-ESP, the NHLBI "Grand Opportunity" Exome Sequencing Project; ExAC, Exome Aggregation Consortium

**Additional file 2: Table S2.** Demographic and clinical characteristics of 117 advanced NSCLC patients from TCGA.

| Variables | Lung adenocarcinoma | | Lung squamous cell carcinoma | |
| --- | --- | --- | --- | --- |
|  | Patients | Deaths | Patients | Deaths |

| Age |
| --- |

| > 65 | 17 | 20 | 12 | 9 |
| --- | --- | --- | --- | --- |
| ≤ 65 | 22 | 16 | 15 | 6 |

| Sex |
| --- |

| Female | 20 | 21 | 7 | 2 |
| --- | --- | --- | --- | --- |
| Male | 19 | 15 | 20 | 13 |

| Smoking |
| --- |

| Ever | 6 | 5 | 1 | 1 |
| --- | --- | --- | --- | --- |
| Never | 33 | 31 | 26 | 14 |

| Metastasis |
| --- |

| M0 | 17 | 26 | 20 | 13 |
| --- | --- | --- | --- | --- |
| M1 or MX | 21 | 10 | 7 | 1 |
