# Supplementary figures and images for: A genetic variant in long non-coding RNA MALAT1 associated with survival outcome among patients with advanced lung adenocarcinoma: a survival cohort analysis
Source: BMC Cancer. 2017 Mar 3;17:167. doi: 10.1186/s12885-017-3151-6 (PMC5335789; doi:10.1186/s12885-017-3151-6)

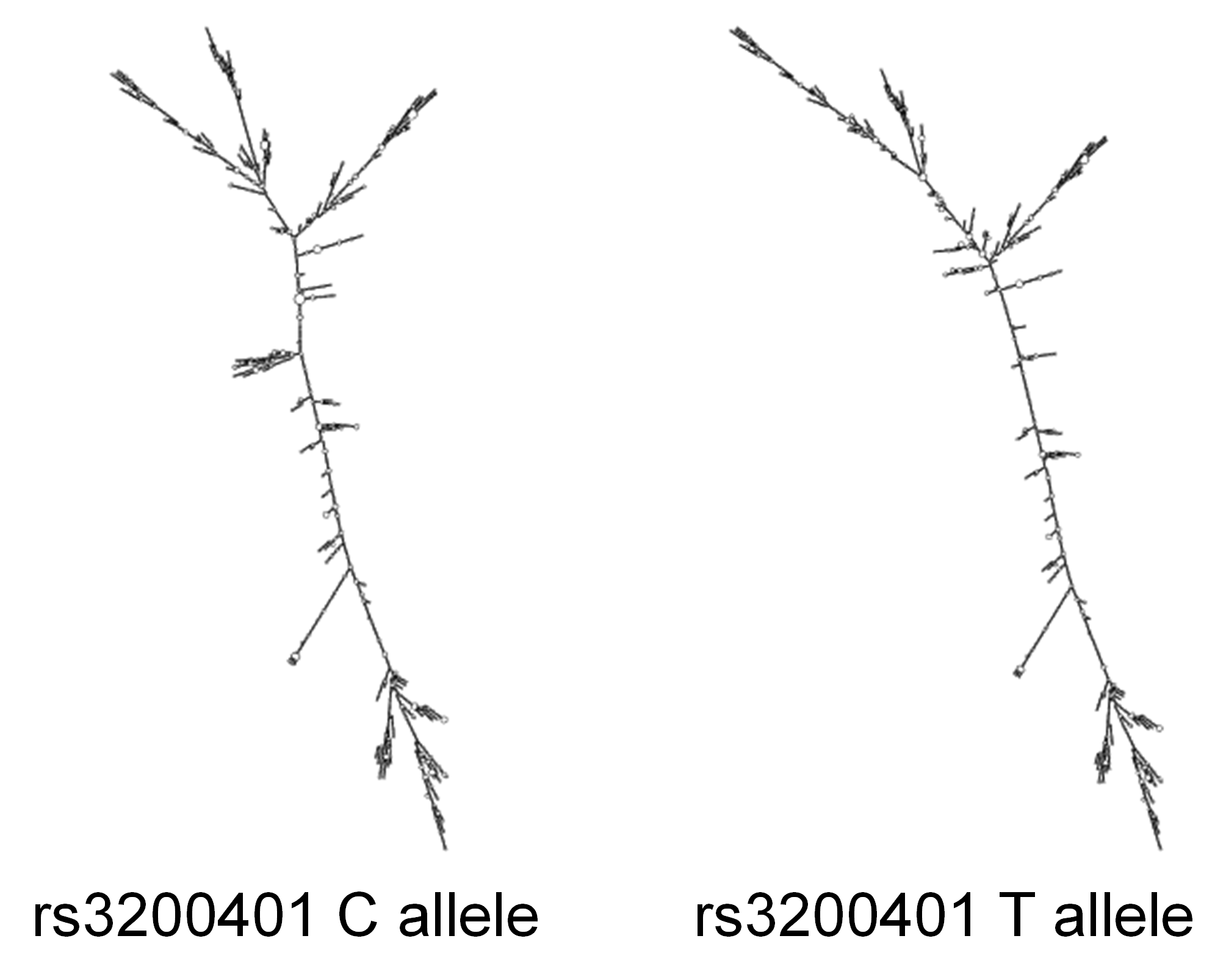

Supplement: Additional file 2: Figure S1. — Predicted secondary structures of lncRNA MALAT1. C > T variation of rs3200401 caused 1.62 kcal/mol minimal free energy (MFE, ΔG) change, which may alter structural features of MALAT1, resulting in weaken interaction between MALAT1 and its binding protein SRSF2. (TIF 215 kb) [file 12885_2017_3151_MOESM2_ESM.tif]
